# Association between anthropometric indicators of obesity and cardiovascular risk factors among adults in Shanghai, China

**DOI:** 10.1186/s12889-019-7366-0

**Published:** 2019-08-02

**Authors:** Yue Zhang, Yi’an Gu, Na Wang, Qi Zhao, Nawi Ng, Ruiping Wang, Xiaoyan Zhou, Yonggen Jiang, Weibing Wang, Genming Zhao

**Affiliations:** 1 Department of Epidemiology, School of Public Health, Key Laboratory of Public Health Safety of Ministry of Education, Fudan University, 138 Yi Xue Yuan Road, Shanghai, 200032 China; 2 Department of Epidemiology, Columbia University, New York, USA; 3 Songjiang Disease Prevention and Control Center, Shanghai, 201600 China; 4 Department of Public Health and Clinical Medicine, Unit for Demography and Aging Research at Umeå University, Umeå, Sweden

**Keywords:** Cardiovascular diseases, Obesity, Anthropometric indices, Risk factors

## Abstract

**Background:**

To determine the optimal cut-off values and evaluate the associations of body mass index (BMI), waist circumference (WC) and waist-height ratio (WHtR) with cardiovascular disease (CVD) risk factors.

**Methods:**

A large-scale cross-sectional survey was conducted among 35,256 adults aged 20–74 years in Shanghai between June 2016 and December 2017. Receiver operating characteristic (ROC) analyses were conducted to assess the optimal cut-off anthropometric indices of CVD risk factors including hypertension, diabetes, dyslipidemia and hyperuricemia. Multivariate Logistic regression models were preformed to evaluate the odds ratio of CVD risk factors.

**Results:**

The area under the curve (AUC) of WHtR was significantly greater than that of BMI or WC in the prediction of hypertension and diabetes, and AUCs were higher in women than men. The optimal cut-off values of WHtR were approximately 0.51 in both sexes, while the cut-off values of BMI and WC were higher for men compared with women. The optimal cutoff values of BMI and WC varied greatly across different age groups, but the difference in WHtR was relatively slight. Among women, the optimal threshold of anthropometric indices appeared to increase with age for hypertension and diabetes. The odds ratio between anthropometric indices and CVD risk factors were attenuated with age. WHtR had the greatest odds ratio for CVD risk factors among adults under 60 years old except for women with hypertension, while among 60–74 years, BMI yielded the greatest odds ratio in terms of all CVD outcomes except for women with diabetes.

**Conclusions:**

WHtR had the best performance for discriminating hypertension and diabetes and potentially be served as a standard screening tool in public health. The associations between three anthropometric indices and CVD risk factors differed by sex and decreased with age. These findings indicated a need to develop age- and gender-specific difference and make effective strategies for primary prevention of CVDs.

**Electronic supplementary material:**

The online version of this article (10.1186/s12889-019-7366-0) contains supplementary material, which is available to authorized users.

## Background

Cardiovascular diseases (CVDs) are serious circulatory diseases that endanger human health and the main cause of mortality worldwide [1, 2]. The World Health Organization (WHO) reports that about 17.9 million people died from CVDs, accounting for 31% of global deaths [3]. It is established that adults characterized by excessive adipose tissue and ectopic fat stores have a higher risk of CVDs [4, 5]. Long-standing evidence has demonstrated the linkage between obesity and increased risk of hypertension, type-2 diabetes mellitus (T2DM) and dyslipidemia, which are major risk factors for CVDs [6, 7]. However, the definition of obesity remains controversial and discrepancies in definitions may lead to inaccurate assessment for CVD risk factors. Body mass index (BMI) is generally recognized anthropometric index of obesity, and numbers of epidemiological studies have confirmed that BMI can predict CVD risk factors [8]. Nevertheless, the neglect of body fat distribution is considered as a typical limitation of BMI. Waist circumference (WC) is recommended as an effective index for measuring the distribution of visceral fat and closely related to cardiometabolic risk [9, 10]. But the variation of WC thresholds across country, ethnic groups, sex and age remains an important issue to be taken into account [11]. Recently, compared with WC, waist-height ratio (WHtR) can account for differences in height, which is easy to measure and variation slightly, and has been proposed a superior predictor of CVD risk factors [12, 13]. To sum up, with regard to the best anthropometric indicator for CVD risk factors still remains inconsistent.

As we all know, WHO recommend that BMI ≥ 30 kg/m^2^ was defined as general obesity and WC ≥ 102 cm in male and ≥ 88 cm in female was identified as central obesity [14]. But more evidences have shown that WHO recommended cutoff values may not be able to detect those with CVD risk factors in Asian populations [15, 16]. Compared with Western counterparts, the same level of BMI and WC among Asians may have a higher percentage of body fat due to racial disparity of physical characteristics [17, 18]. It can be seen that the threshold for obesity does not apply to any population. Although some studies have been conducted to assess which anthropometric indicators are more strongly related to CVD risk factors in other provinces in China, controversies still existed [19, 20]. Shanghai is the most populous and economically developed city, the process of urbanization, the degree of aging, socioeconomic characteristics and lifestyle of population are quite different from those of other districts in China. Moreover, few studies assessed the discriminatory power of anthropometric indices for CVD risk factors by sex and age subgroups. It is very necessary to conduct large-scale surveys in our population to explore the optimal cut-off values for CVD risk factors. Thus, the objective of our study is to determine the optimal cut-off points of BMI, WC and WHtR for CVD risk factors (hypertension, T2DM, dyslipidemia and hyperuricemia) and explore the associations between anthropometric measures and CVD risk factors stratified by sex and age among adults in Shanghai, China.

## Methods

### Study population

From June 2016 to December 2017, we conducted a population-based cross-sectional survey in Songjiang District, Shanghai. A multistage stratified sampling method was used to recruit participants. At stage one, we purposively selected four study areas in Songjiang District stratified by geographic region and economic levels, which included two urban communities (Zhongshan and Xinqiao), one urban-rural mixed region (Sheshan) and one rural district (Maogang). At stage two, nine and eighteen street districts were selected from Xinqiao and Zhongshan randomly. Four neighborhood committees were randomly selected from Sheshan, and sixteen administrative villages were selected from Maogang. At stage three, individuals aged 20–74 years were selected randomly from each of administrative units in stage two. We totally recruited 37,670 permanent participants who had been living in Songjiang district for 5 years or longer. Residents with disabilities, terminal illness or perceptual impairment were excluded. After exclusion of individuals who lacked data in any of the variables, 35,256 residents (a response rate was 93.6%) were left for final analysis.

### Data collection

#### Questionnaire interview

The electronic questionnaire (Additional file 2) consists of questions on demographic characteristics (e.g., sex, age, education level), health-related behaviors (e.g., current smoking and drinking status), and history of self-reported chronic diseases (e.g., hypertension, diabetes). Education was recorded as completed years of schooling and categorized to three categories of ≤6 years (primary school and lower), 7–12 years (middle and high school) and > 12 years (college and above). For smoking, participants were asked two questions: “Have you ever smoked at least one cigarette every day for more than six months (yes, no)?” and “What is your smoking status now (current and non-current)? (for individuals who answered ‘yes’)?” We defined current smoking as having smoked every day for more than six months and currently smoking cigarettes. For alcohol consumption, participants were asked two questions: “Have you consumed alcohol at least 3 times a week for more than 6 months (yes, no)?” and “Have you consumed alcohol during the past year (for individuals who answered ‘yes’)?” In order to ensure quality stringently, all subject information was electronically coded and stored in the database. At each stage of the investigation, two quality controllers were arranged to check data collection, make logical judgment and correct the data with poor quality in time.

#### Measurements

Anthropometric measurements were conducted in participants wearing light clothing with no footwear. All the measurements were performed by community licensed physicians according to the standard protocol. BMI was calculated as weight (accurate to 0.1 kg) in kilograms divided by height (accurate to 0.1 cm) in meters squared (kg/m^2^). WC was measured at the mid-point between the iliac crest and the last rib while the subject was at minimal respiration by a flexible tape. WHtR was calculated as WC (cm) divided by height (cm). Blood pressure was measured on the right arm in a sitting position using a digital sphygmomanometer after five minutes of rest. Three measurements were taken and mean values was recorded.

#### Laboratory assay

Blood samples were collected into vacuum tubes in the morning after an overnight fast. The specimens were obtained and stored at − 80 °C freezer within less than 6 h and thereafter transported to the Shanghai Dian Diagnostics Co Ltd. Serum total cholesterol (TC), triglycerides (TG), low-density lipoprotein cholesterol (LDL-C) and high-density lipoprotein cholesterol (HDL-C) were measured using enzyme colorimetry (Roche COBAS C501 automatic biochemical analyzer). Fasting plasma glucose (FPG) were measured using Glycokinase method (Roche P800 automatic biochemical analyzer). Glycated hemoglobin (HbA1c) were measured using high pressure liquid chromatography (TOSOH G8, Automatic hemoglobin A1c analyzer). Uric acid (UA) was measured using colorimetry (Roche c702 automatic biochemical analyzer).

#### Definition of CVD risk factors

*Hypertension* was defined as systolic blood pressure (SBP) ≥ 140 mmHg and/or a diastolic blood pressure (DBP) ≥ 90 mmHg or self-reported history of hypertension. *Diabetes* was defined by American Diabetes Association (ADA) criteria: as HbA1c ≥ 6.5% or FPG ≥ 7.0 mmol/L or self-reported history of diabetes [21]. *Dyslipidemia* was defined as at least one of the following characteristics: high TC (TC ≥ 6.22 mmol/L); high LDL-C (LDL-C ≥ 4.14 mmol/L); low HDL-C (HDL-C < 1.04 mmol/L) or hypertriglyceridemia (TG ≥ 2.26 mmol/L) [22] . *Hyperuricemia* was defined as uric acid ≥7.0 mg/dl (416.0 μmol/L) in men and ≥ 6.0 mg/dl (357.0 μmol/L) in women [23].

### Statistical analysis

Continuous variables are represented by mean (standard deviation (SD)) and categorical variables by percentages. Cut-off value was determined between each anthropometrical index and CVD risk factors by the receiver operating characteristic (ROC) curve, which was quantified by the area under ROC curve (AUC). The Youden’s index (sensitivity+specificity-1) was used to determine the optimal cutoff point of each index. Z test was used to determine differences between two AUCs: $\mathrm{Z}=\frac{\ {AUC}_A-{AUC}_B}{\sqrt{{S_{EA}}^2+{S_{EB}}^2}}$, (S_E_ is standard error, Z > 1.96, *p* < 0.05). Multivariate logistic regression analysis was performed to estimate the association between each SD increase of anthropometrical index and CVD risk factors after adjusting for age, educational level, current smoking and drinking by sex. The adjusted odds ratios (ORs) are presented with 95% confidence interval (CIs). All the analyses were conducted using SPSS version 23 software (SPSS Inc., Chicago, IL, USA). Two-tailed *p* < 0.05 was considered to be statistically significant.

### Ethical approval

The Shanghai cohort study protocol was approved by the ethical review committee of School of Public Health, Fudan University (IRB approval number 2016-04-0586). Written informed consent was obtained from all study participants prior to the study.

## Results

The basic characteristics and prevalence of CVD risk factors are presented in Table 1. Compared with women, men had a significantly higher mean BMI, WC, SBP, DBP, FPG, Hb1Ac, TG and UA, but lower mean WHtR, TC, LDL-C and HDL-C. Men had higher prevalence of hypertension (56.05% vs. 49.72%), diabetes (15.19% vs. 13.71%), dyslipidemia (34.35% vs.25.74%) and hyperuricemia (16.63% vs.8.86%) than women. Only 0.68 and 0.22% of the women were current drinkers and smokers, respectively.

**Table 1:** The basic characteristics and prevalence of CVD risk factors in Songjiang district, Shanghai

	Men(*n* = 14,311)	Women(*n* = 20,945)	*p* value
Age (years)	57.22 ± 11.28	55.58 ± 11.14	< 0.001
Education, n (%)			< 0.001
0–6 years	5557 (38.83)	10,798 (51.55)	
7–12 years	7875 (55.03)	8865 (42.33)	
> 12 years	879 (6.14)	1282 (6.12)	
Current smoking, n (%)	6921 (48.36)	46 (0.22)	< 0.001
Current drinking, n (%)	4184 (29.24)	143 (0.68)	< 0.001
BMI (kg/m^2^)	24.73 ± 3.22	24.19 ± 3.43	< 0.001
WC (cm)	84.64 ± 8.86	79.68 ± 9.29	< 0.001
WHtR	0.50 ± 0.05	0.51 ± 0.06	< 0.001
SBP (mmHg)	133.91 ± 18.32	133.37 ± 20.13	0.012
DBP (mmHg)	81.88 ± 10.41	78.81 ± 10.39	< 0.001
FPG (mmol/L)	5.11 ± 1.49	5.06 ± 1.33	< 0.001
Hb1Ac (%)	5.81 ± 0.91	5.77 ± 0.82	< 0.001
TC (mmol/L)	4.78 ± 0.92	5.04 ± 0.94	< 0.001
TG (mmol/L)	1.74 ± 1.40	1.59 ± 1.09	< 0.001
HDL-C (mmol/L)	1.30 ± 0.34	1.48 ± 0.35	< 0.001
LDL-C (mmol/L)	2.70 ± 0.82	2.83 ± 0.85	< 0.001
UA (mmol/L)	348.02 ± 79.43	271.85 ± 65.78	< 0.001
Hypertension, n (%)	8021 (56.05)	10,414 (49.72)	< 0.001
Diabetes, n (%)	2174 (15.19)	2871 (13.71)	< 0.001
Dyslipidemia, n (%)	4916 (34.35)	5392 (25.74)	< 0.001
Hyperuricemia, n (%)	2380 (16.63)	1856 (8.86)	< 0.001

Data are Means ± standard deviation SD or n (%)

*Abbreviations*: *BMI* body mass index, *WC* waist circumference, *WHtR* waist-to-height ratio, *FPG* fasting blood glucose, *SBP* systolic blood pressure, *DBP* diastolic blood pressure, *TC* total cholesterol, *TG* triglycerides, *HDL-C* high-density lipoprotein cholesterol, *LDL-C* low-density lipoprotein cholesterol, *HbA1c* hemoglobin, *UA* uric acid

Table 2 shows the correlation between anthropometric index and CVD risk factors. BMI, WC and WHtR were significantly correlated with the CVD risk factors in both sexes (*p* < 0.001). Compared with BMI and WC, WHtR had the highest correlation with SBP, FPG, Hb1Ac, TC and LDL-C for both men and women.

**Table 2:** Pearson’s correlation coefficients between anthropometric indices and CVD risk factors among men and women

	Men			Women		
	BMI	WC	WHtR	BMI	WC	WHtR
SBP	0.25 (0.24,0.27)	0.23 (0.21,0.24)	0.27 (0.25,0.28)	0.30 (0.29,0.31)	0.32 (0.31,0.34)	0.36 (0.34,0.37)
DBP	0.26 (0.25,0.28)	0.22 (0.21,0.24)	0.22 (0.20,0.23)	0.25 (0.24,0.27)	0.21 (0.20,0.22)	0.21 (0.20,0.23)
FPG	0.10 (0.09,0.12)	0.09 (0.07,0.10)	0.11 (0.09,0.12)	0.15 (0.14,0.17)	0.14 (0.13,0.16)	0.16 (0.15,0.17)
Hb1Ac	0.15 (0.14,0.17)	0.15 (0.13,0.17)	0.18 (0.16,0.19)	0.23 (0.21,0.24)	0.25 (0.24,0.27)	0.28 (0.27,0.29)
TC	0.07 (0.06,0.09)	0.07 (0.06,0.09)	0.08 (0.06,0.09)	0.09 (0.08,0.10)	0.12 (0.11,0.13)	0.14 (0.13,0.15)
TG	0.23 (0.22,0.25)	0.23 (0.22,0.25)	0.20 (0.19,0.22)	0.21 (0.20,0.22)	0.22 (0.21,0.23)	0.22 (0.21,0.24)
HDL-C	−0.32(−0.33, −0.30)	−0.30(−0.31, −0.28)	−0.26(−0.27, −0.24)	−0.25(−0.27, −0.21)	−0.23(−0.25, −0.19)	−0.21(−0.23, −0.18)
LDL-C	0.04 (0.02,0.05)	0.03 (0.01,0.05)	0.04 (0.03,0.06)	0.08 (0.07,0.09)	0.10 (0.09,0.12)	0.12 (0.10,0.13)
UA	0.23 (0.21,0.24)	0.22 (0.20,0.23)	0.19 (0.18,0.21)	0.31 (0.30,0.33)	0.29 (0.28,0.30)	0.29 (0.28,0.30)

Data are correlation coefficients and 95% confidence intervals

All correlation coefficients are significant at *p* < 0.001

CVD: cardiovascular diseases. Other abbreviations see Table 1

**Fig. 1:**
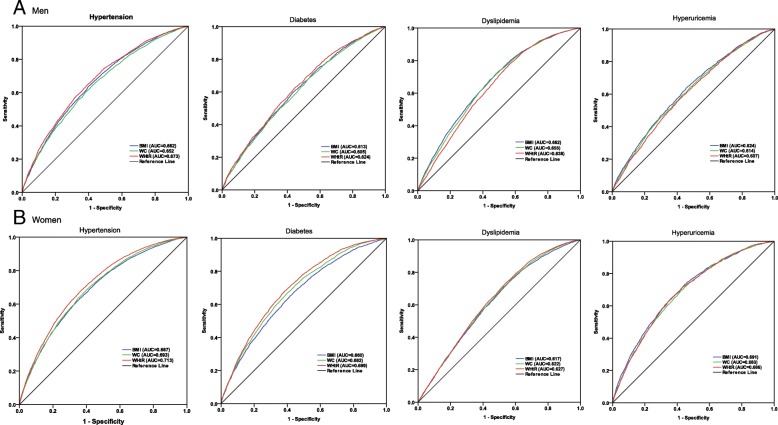
ROC curves of the anthropometric indices for hypertension, diabetes, dyslipidemia and hyperuricemia in men and women (**a**: Men; **b**: Women)

The ROC curves and AUCs of BMI, WC and WHtR for hypertension, diabetes, dyslipidemia, and hyperuricemia by sex are displayed in Fig. 1 and Table 3. Regarding hypertension and diabetes, the AUCs for WHtR were higher than WC among men, and were higher than BMI among women. No significant difference in AUCs was found between three anthropometric indices and dyslipidemia and hyperuricemia in either men or women. The AUCs for anthropometric indices stratified by age groups are summarized in Additional file 1: Table S1. The AUCs between three anthropometric indices and CVD outcomes tended to decrease with age.

**Table 3:** AUC for various anthropometric indices and CVD risk factors in men and women

Risk factor	BMI	WC	WHtR
Men			
Hypertension	0.662 (0.653,0.671)	0.650 (0.641,0.659)	0.673 (0.665,0.682) *
Diabetes	0.613 (0.600,0.625)	0.605 (0.592,0.618)	0.624 (0.612,0.637) *
Dyslipidemia	0.662 (0.653,0.671)	0.655 (0.645,0.664)	0.638 (0.629,0.648)
Hyperuricemia	0.624 (0.612,0.636)	0.614 (0.601,0.626)	0.607 (0.595,0.619)
Women			
Hypertension	0.687 (0.680,0.694)	0.693 (0.686,0.700)	0.713 (0.706,0.719) * ^#^
Diabetes	0.660 (0.650,0.671)	0.682 (0.672,0.692) ^#^	0.699 (0.690,0.709) ^#^
Dyslipidemia	0.617 (0.608,0.625)	0.622 (0.613,0.630)	0.627 (0.619,0.635)
Hyperuricemia	0.691 (0.679,0703)	0.683 (0.670,0.695)	0.686 (0.674,0.698)

*AUC* area under the ROC curve, *BMI* body mass index, *WC* waist circumference, *WHtR* waist-to-height ratio

*p < 0.05, compared to WC

#*p* < 0.05, compared to BMI

Table 4 shows the optimal cutoff points of three anthropometric indices based on ROC analysis for CVD risk factors. The optimal BMI cut-off values varied from 24.39 to 24.93 kg/m^2^ in men, and 23.09–24.16 kg/m^2^ in women. The optimal WC cut-off values varied from 83.38 to 85.68 cm in men, and 77.68–80.95 cm in women for all the CVD outcomes. The optimal WHtR cut-off values varied from 0.49 to 0.52 in men, and 0.49 to 0.53 in women. With regard to BMI and WC, the optimal cut-off values were mostly higher for men compared with women. Furthermore, the optimal cutoff values of BMI and WC varied across different age groups, while the difference in WHtR was relatively smaller (Additional file 1: Table S2 and S3). For women, the optimal cutoffs of BMI, WC and WHtR for hypertension and diabetes were higher in older age groups (60–74 years).

**Table 4:** Optimal cut-off value for anthropometric indices predictive of CVD risk factors

	BMI				WC				WHtR			
	Cutoff	Sen	Spe	YI	Cutoff	Sen	Spe	YI	Cutoff	Sen	Spe	YI
Men												
Hypertension	24.39	63.75	60.20	0.24	83.95	64.88	57.22	0.22	0.51	65.33	60.16	0.26
Diabetes	24.93	59.89	56.51	0.16	83.95	68.17	47.17	0.15	0.52	62.42	55.64	0.18
Dyslipidemia	24.45	68.07	55.74	0.24	83.38	71.38	51.99	0.23	0.49	77.62	43.75	0.21
Hyperuricemia	24.70	64.35	53.97	0.18	85.68	59.45	56.83	0.16	0.52	58.66	56.76	0.15
Women												
Hypertension	23.95	63.30	64.10	0.27	79.75	63.77	64.78	0.29	0.51	68.30	63.00	0.31
Diabetes	24.16	67.08	56.27	0.23	80.95	67.29	59.47	0.27	0.52	68.09	61.49	0.30
Dyslipidemia	23.09	73.80	44.25	0.18	77.68	71.05	47.40	0.18	0.49	73.98	45.42	0.19
Hyperuricemia	24.16	73.00	55.58	0.29	80.68	68.86	58.04	0.27	0.53	64.39	64.13	0.29

*BMI* body mass index, *WC* waist circumference, *WHtR* waist-to-height ratio, *Sen (%)* sensitivity;

Spe (%): specificity; YI: Youden’s index

**Fig. 2:**
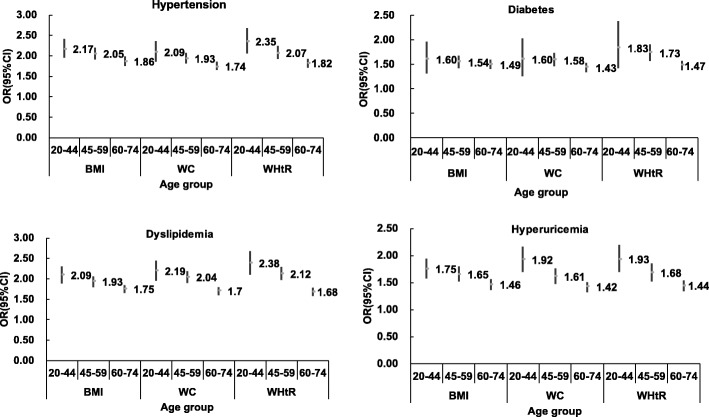
Odds ratios (ORs) and 95% CI for CVD risk factors corresponding to one SD increase in BMI, WC and WHtR by age groups in men

**Fig. 3:**
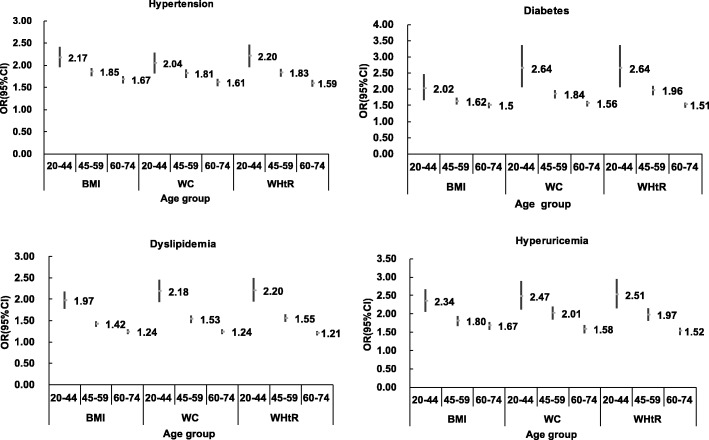
Odds ratios (ORs) and 95% CI for CVD risk factors corresponding to one SD increase in BMI, WC and WHtR by age groups in women

Table 5 shows adjusted ORs of CVD risk factors with one SD increase of BMI, WC and WHtR. WHtR had the highest ORs for diabetes, dyslipidemia and hyperuricemia in men, while WC yielded the greatest ORs in women. BMI gave the relatively higher ORs for hypertension in both sexes. Figures 2 and 3 shows the ORs for CVD risk factors according to one SD increase in anthropometric measures categorized across age groups by sex. Results indicated that association between anthropometric indices and CVD risk factors were attenuated with age. WHtR had relatively higher ORs in the group aged 20–44 and 45–59 years except hypertension in women, while among 60–74 years the ORs for BMI were highest in terms of all CVD outcomes except diabetes in women.

**Table 5:** Odd ratios and 95% CI for CVD risk conditions corresponding to one SD increase in BMI, WC and WHtR

	BMI		WC		WHtR	
	OR	95%CI	OR	95%CI	OR	95%CI
Men ^a^						
Hypertension	**1.98**	**1.90–2.06**	1.85	1.78–1.93	1.97	1.88–2.06
Diabetes	1.52	1.45–1.60	1.49	1.42–1.57	**1.57**	**1.49–1.66**
Dyslipidemia	1.87	1.79–1.94	1.88	1.80–1.96	**1.92**	**1.83–2.00**
Hyperuricemia	1.57	1.50–1.65	1.55	1.48–1.63	**1.58**	**1.50–1.66**
Women ^b^						
Hypertension	**1.80**	**1.75–1.87**	1.74	1.68–1.80	1.74	1.69–1.80
Diabetes	1.56	1.50–1.63	**1.69**	**1.61–1.76**	1.68	1.61–1.75
Dyslipidemia	1.37	1.33–1.42	**1.42**	**1.37–1.47**	1.40	1.35–1.45
Hyperuricemia	1.79	1.71–1.87	**1.80**	**1.71–1.89**	1.74	1.65–1.82

*CI* confidence interval, *BMI* body mass index, *WC* waist circumference, *WHtR* waist-to-height ratio

^a^ Adjusted for age, current smoking, current drinking and educational level

^b^ Adjusted for age and educational level

Anthropometric measure with the highest significant OR value in bold

## Discussion

This study evaluated the optimal cut-off values and discriminatory ability of BMI, WC and WHtR for CVD risk factors among adults in Shanghai. Our results indicated that BMI cutoff values were comparable with those of the recommended cutoff by Working Group on Obesity in China (WGOC), but WC cut-off values were relatively lower. Results from previous studies demonstrated that WC threshold of about 80–85 cm for men and 75–80 cm for women [9, 24], which were similar to our results. One study of deriving the optimal WC threshold for cardiometabolic risk in sub-Saharan Africa found that the optimal cutoff at increased cardiometabolic risk was lower (81.2 cm) than current recommend guidelines in men [25]. Several prospective studies have proposed that WHtR cutoff value of 0.50 could be applied to different populations [26]. Our study supported this view that the cutoff of WHtR was approximately 0.51 for CVD risk factors. One cardiovascular study among Filipino-American women revealed the best cutoff point of WHtR was 0.50 and only 1% of patients with hypertension and 0% of patients with diabetes cannot be detected [27]. The cutoffs of WHtR were 0.52–0.53 for identifying several CVD risk factors in both sexes in Jinan, China [28]. Another study suggested that the optimal threshold of WHtR was 0.48–0.51 for men and 0.47–0.50 for women for predicting cardiometabolic risk in Taiwan [29]. The slight variations of WHtR made it likely to be applied to public health practice as a simple screening tool. The reason for small changes of threshold of WHtR was probably differences in sample size, age group or statistical methods.

In this study, WHtR tended toward the best performance for discriminating hypertension and diabetes, which was in agreement with other studies [30, 31]. However, the differences in AUCs between anthropometric indicators and dyslipidemia and hyperuricemia were small and 95%CIs were overlapping. Hence, BMI, WC, and WHtR may be comparable in their ability to identify these two CVD risk factors. Apart from this, due to the units of each anthropometric indicator were different, it is inappropriate to directly compare OR values. Therefore, we estimated the ORs in terms of each SD increase of the anthropometric measure, and found that WHtR or WC may have better association except for hypertension based on point estimates of associations in both sexes. Previous epidemiology studies have shown that central obesity indicators such as WC and WHtR are more closely associated with CVD risk factors than BMI, which was consistent with our results [32, 33]. This may be because ectopic fat deposition triggers pathological metabolic reactions, which increased the risk of metabolic diseases [34]. It is noteworthy that in our study, the AUCs decreased with age, indicating that the ability to recognize CVD risk factors gradually weakened with age. The association was strongest between anthropometric indices and CVD risk factors among 20–44 age group regardless of sex. A recent study on the association between obesity and hypertension among Chinese elderly provided evidence that anthropometric indicators varied with age [35]. Other studies have also demonstrated this similar age-specific difference [36, 37], possibly because that older people tend to lose muscle mass and have more concentrated fat distribution with age [38]. Thus, targeted weight management will benefit greatly among young population. Additionally, the present study also demonstrated among aged 60–74 years the ORs of BMI were highest in terms of all CVD outcomes except for diabetes in women, but WHtR had relatively higher ORs for people under 60 years except women with hypertension. The potential mechanism is unclear, thus more researches are needed to explore the potential age difference for elucidating the role of age in the pathogenesis of obesity and CVD risk factors.

Strengths of this study include a large population-based sample size and extensive CVD risk factor data available for study participants. This study is the first representative sample containing most age groups to determine the optimal cut-off values for CVD risk factors among Shanghai adults. The present study also has some notable limitations. Firstly, cross-sectional study cannot estimate the causality between anthropometric measures and CVD risk factors. In order to confirm the results of this study, further prospective cohort studies are needed. In addition, as our study was conducted only in Shanghai, it limits the generalizability of our results to other regions in China. Secondly, diet, stress, treatment and genetics are known confounders for anthropometric measures of obesity and CVD risk factors, due to unavailable of these data, we did not consider these variables in the analysis. Finally, we could not account for the relationship between more measures of obesity (hip circumference, waist-to-hip ratio, body adiposity index along with others) and CVD risk factors because of limited data.

## Conclusions

In summary, our data suggested the optimal cut-off of WHtR was approximately 0.51 for CVD risk factors and it tended to perform best in discriminating hypertension and diabetes in both sexes in Shanghai. The optimal cutoff values of BMI and WC varied greatly for CVD risk factors across different sex and age groups, while the difference in WHtR was relatively smaller. The discriminating power of anthropometric indictors and associations with CVD risk factors were attenuated with age. Thus, weight management and control should be strengthened at a young age for primary prevention of cardiovascular diseases.

## Additional files

Additional file 1: **Table S1.** AUCs for anthropometric indices and CVD risk factors in men and women by age groups. **Table S2.** Optimal cut-off values for BMI, WC and WHtR that are predictive of CVD risk factors in men by age groups. **Table S3.** Optimal cut-off values for BMI, WC and WHtR that are predictive of CVD risk factors in women by age groups. (DOCX 30 kb)

Additional file 2: Questionnaire. (DOCX 57 kb)

## Data Availability

The datasets generated during and/or analyzed during the current study are not publicly available due to confidentiality of data and subsequent research, but are available from the corresponding author on reasonable request.

## Abbreviations

AUC: Area under the curve
BMI: Body mass index
CI: Confidence interval
CVD: Cardiovascular diseases
DBP: Diastolic blood pressure
FPG: Fasting blood glucose
HbA1c: Hemoglobin
HDL-C: High-density lipoprotein cholesterol
LDL-C: Low-density lipoprotein cholesterol
ROC: Receiver operating characteristic
SBP: Systolic blood pressure
TC: Total cholesterol
TG: Triglycerides
UA: Uric acid
WC: Waist circumference
WHtR: Waist-to-height ratio

## References

[1] Zhou M, Wang H, Zhu J, Chen W, Wang L, Liu S, Li Y, Wang L, Liu Y, Yin P (2016). Cause-specific mortality for 240 causes in China during 1990–2013: a systematic subnational analysis for the global burden of disease study 2013. Lancet.

[2] Roth GA, Johnson C, Abajobir A, Abd-Allah F, Abera SF, Abyu G, Ahmed M, Aksut B, Alam T, Alam K (2017). Global, regional, and national burden of cardiovascular diseases for 10 causes, 1990 to 2015. J Am Coll Cardiol.

[3] Naghavi M, Abajobir AA, Abbafati C, Abbas KM, Abd-Allah F, Abera SF, Aboyans V, Adetokunboh O, Afshin A, Agrawal A (2017). Global, regional, and national age-sex specific mortality for 264 causes of death, 1980–2016: a systematic analysis for the global burden of disease study 2016. Lancet.

[4] Neeland IJ, Poirier P, Després J-P (2018). Cardiovascular and metabolic heterogeneity of obesity: clinical challenges and implications for management. Circulation.

[5] Abraham TM, Pedley A, Massaro JM, Hoffmann U, Fox CS (2015). Association between visceral and subcutaneous adipose depots and incident cardiovascular disease risk factors. Circulation.

[6] Emerging Risk Factors Collaboration (2011). Separate and combined associations of body-mass index and abdominal adiposity with cardiovascular disease: collaborative analysis of 58 prospective studies. Lancet.

[7] Luo J, Hendryx M, Laddu D, Phillips LS, Chlebowski R, LeBlanc ES, Allison DB, Nelson DA, Li Y, Rosal MC (2019). Racial and ethnic differences in anthropometric measures as risk factors for diabetes. Diabetes Care.

[8] Berrington de Gonzalez A, Hartge P, Cerhan JR, Flint AJ, Hannan L, RJ MI, Moore SC, Tobias GS, Anton-Culver H, Freeman LB (2010). Body-mass index and mortality among 1.46 million white adults. New Engl J Med.

[9] Zeng Q, He Y, Dong S, Zhao X, Chen Z, Song Z, Chang G, Yang F, Wang Y (2014). Optimal cut-off values of BMI, waist circumference and waist: height ratio for defining obesity in Chinese adults. Br J Nutr.

[10] See R, Abdullah SM, McGuire DK, Khera A, Patel MJ, Lindsey JB, Grundy SM, De Lemos JA (2007). The association of differing measures of overweight and obesity with prevalent atherosclerosis: the Dallas heart study. J Am Coll Cardiol.

[11] Misra A, Wasir JS, Vikram NK (2005). Waist circumference criteria for the diagnosis of abdominal obesity are not applicable uniformly to all populations and ethnic groups. Nutrition.

[12] Dong J, Ni Y-Q, Chu X, Liu Y-Q, Liu G-X, Zhao J, Yang Y-B, Yan Y-X (2016). Association between the abdominal obesity anthropometric indicators and metabolic disorders in a Chinese population. Public Health.

[13] Peer N, Lombard C, Steyn K, Levitt N (2018). Utility of waist-to-height ratio as an Indicator of cardio-metabolic risk compared with routinely used adiposity indices. J Hypertens.

[14] Grundy SM, Brewer HB, Cleeman JI, Smith SC, Lenfant C (2004). Definition of metabolic syndrome: report of the National Heart, Lung, and Blood Institute/American Heart Association conference on scientific issues related to definition. Circulation.

[15] Shalini D, Ponnalagu O, Bi X, Henry CJ (2019). Is waist circumference more strongly associated with metabolic risk factors than waist-to-height ratio in Asians?. Nutrition.

[16] Ramezankhani A, Ehteshami-Afshar S, Hasheminia M, Hajebrahimi MA, Azizi F, Hadaegh F (2018). Optimum cutoff values of anthropometric indices of obesity for predicting hypertension: more than one decades of follow-up in an Iranian population. J Hum Hypertens.

[17] Lear SA, Humphries KH, Kohli S, Chockalingam A, Frohlich JJ, Birmingham CL (2007). Visceral adipose tissue accumulation differs according to ethnic background: results of the multicultural community health assessment trial (M-CHAT). Am J Clin Nutr.

[18] Chan JC, Malik V, Jia W, Kadowaki T, Yajnik CS, Yoon K-H, Hu FB (2009). Diabetes in Asia: epidemiology, risk factors, and pathophysiology. Jama.

[19] Thomas GN, Ho SY, Lam KS, Janus ED, Hedley AJ, Lam TH (2004). Impact of obesity and body fat distribution on cardiovascular risk factors in Hong Kong Chinese. Obes Res.

[20] Yu J, Tao Y, Tao Y, Yang S, Yu Y, Li B, Jin L (2016). Optimal cut-off of obesity indices to predict cardiovascular disease risk factors and metabolic syndrome among adults in Northeast China. BMC Public Health.

[21] American Diabetes Association (2019). 2. Classification and Diagnosis of Diabetes: Standards of Medical Care in Diabetes—2019. Diabetes Care.

[22] Pan L, Yang Z, Wu Y, Yin R-X, Liao Y, Wang J, Gao B, Zhang L (2016). The prevalence, awareness, treatment and control of dyslipidemia among adults in China. Atherosclerosis.

[23] Liu H, Zhang X-M, Wang Y-L, Liu B-C (2014). Prevalence of hyperuricemia among Chinese adults: a national cross-sectional survey using multistage, stratified sampling. J Nephrol.

[24] Lin W, Lee L, Chen C, Lo H, Hsia H, Liu I, Lin R, Shau W, Huang K (2002). Optimal cut-off values for obesity: using simple anthropometric indices to predict cardiovascular risk factors in Taiwan. Int J Obes.

[25] Ekoru K, Murphy G, Young E, Delisle H, Jerome C, Assah F, Longo-Mbenza B, Nzambi J, On’Kin J, Buntix F (2018). Deriving an optimal threshold of waist circumference for detecting cardiometabolic risk in sub-Saharan Africa. Int J Obes.

[26] Browning LM, Hsieh SD, Ashwell M (2010). A systematic review of waist-to-height ratio as a screening tool for the prediction of cardiovascular disease and diabetes: 0· 5 could be a suitable global boundary value. Nutr Res Rev.

[27] Battie CA, Borja-Hart N, Ancheta IB, Flores R, Rao G, Palaniappan L (2016). Comparison of body mass index, waist circumference, and waist to height ratio in the prediction of hypertension and diabetes mellitus: Filipino-American women cardiovascular study. Prev Med Rep.

[28] Dong X, Liu Y, Yang J, Sun Y, Chen L (2011). Efficiency of anthropometric indicators of obesity for identifying cardiovascular risk factors in a Chinese population. Postgrad Med J.

[29] Li W-C, Chen I-C, Chang Y-C, Loke S-S, Wang S-H, Hsiao K-Y (2013). Waist-to-height ratio, waist circumference, and body mass index as indices of cardiometabolic risk among 36,642 Taiwanese adults. Eur J Nutr.

[30] Rezende AC, Souza LG, Jardim TV, Perillo NB, Araújo YCL, de Souza SG, Sousa ALL, Moreira HG, de Souza WKSB, Peixoto MRG (2018). Is waist-to-height ratio the best predictive indicator of hypertension incidence? A cohort study. BMC Public Health.

[31] Hou X, Chen S, Hu G, Chen P, Wu J, Ma X, Yang Z, Yang W, Jia W (2019). Stronger associations of waist circumference and waist-to-height ratio with diabetes than BMI in Chinese adults. Diabetes Res Clin Pract.

[32] Tran NTT, Blizzard CL, Luong KN, Le Van Truong N, Tran BQ, Otahal P, Nelson M, Magnussen C, Gall S, Van Bui T (2018). The importance of waist circumference and body mass index in cross-sectional relationships with risk of cardiovascular disease in Vietnam. PLoS One.

[33] Choi JR, Koh SB, Choi E (2018). Waist-to-height ratio index for predicting incidences of hypertension: the ARIRANG study. BMC Public Health.

[34] Piché M-E, Poirier P, Lemieux I, Després J-P (2018). Overview of epidemiology and contribution of obesity and body fat distribution to cardiovascular disease: an update. Prog Cardiovasc Dis.

[35] Wang Q, Xu L, Li J, Sun L, Qin W, Ding G, Zhu J, Zhang J, Yu Z, Xie S (2018). Association of anthropometric indices of obesity with hypertension in Chinese elderly: an analysis of age and gender differences. Int J Environ Res Public Health.

[36] Wakabayashi I, Daimon T (2012). Receiver-operated characteristics (ROCs) of the relationships between obesity indices and multiple risk factors (MRFs) for atherosclerosis at different ages in men and women. Arch Gerontol Geriatr.

[37] Cai L, Liu A, Zhang Y, Wang P (2013). Waist-to-height ratio and cardiovascular risk factors among Chinese adults in Beijing. PLoS One.

[38] Cnop M, Havel PJ, Utzschneider KM, Carr DB, Sinha MK, Boyko EJ, Retzlaff BM, Knopp RH, Brunzell JD, Kahn SE (2003). Relationship of adiponectin to body fat distribution, insulin sensitivity and plasma lipoproteins: evidence for independent roles of age and sex. Diabetologia.

