# Supplementary material for: Association between anthropometric indicators of obesity and cardiovascular risk factors among adults in Shanghai, China
Source: BMC Public Health. 2019 Aug 2;19:1035. doi: 10.1186/s12889-019-7366-0 (PMC6679475; doi:10.1186/s12889-019-7366-0)
Supplement: Supplementary file 1 — Table S1. AUCs for anthropometric indices and CVD risk factors in men and women by age groups. Table S2. Optimal cut-off values for BMI, WC and WHtR that are predictive of CVD risk factors in men by age groups. Table S3. Optimal cut-off values for BMI, WC and WHtR that are predictive of CVD risk factors in women by age groups. (DOCX 30 kb) [file 12889_2019_7366_MOESM1_ESM.docx]

| Table S1. AUCs for anthropometric indices and CVD risk factors in men and women by age groups | | | |
| --- | --- | --- | --- |
|  | Age groups | | |
|  | 20-44 | 45-59 | 60-74 |
| **Men** |  |  |  |
| Hypertension |  |  |  |
| BMI | **0.723 (0.698, 0.748)** | **0.678 (0.663, 0.692)** | **0.656 (0.643, 0.669)** |
| WC | 0.694 (0.668, 0.721) | 0.658 (0.644, 0.673) | 0.644(0.631, 0.658) |
| WHtR | 0.710(0.685, 0.736) | 0.664 (0.649, 0.678) | 0.648 (0.634, 0.661) |
| Diabetes |  |  |  |
| BMI | 0.653 (0.595, 0.711) | 0.610 (0.590, 0.631) | **0.611 (0.594, 0.628)** |
| WC | 0.637 (0.578, 0.696) | 0.610 (0.589, 0.630) | 0.595 (0.578, 0.612) |
| WHtR | **0.667 (0.610, 0.723)** | **0.624 (0.604, 0.644)** | 0.600 (0.583, 0.617) |
| Dyslipidemia |  |  |  |
| BMI | **0.712 (0.689, 0.735)** | 0.662 (0.648, 0.677) | **0.646 (0.632, 0.660)** |
| WC | 0.704 (0.680, 0.727) | **0.665 (0.651, 0.680)** | 0.637 (0.623, 0.651) |
| WHtR | 0.710 (0.687, 0.733) | 0.662 (0.647, 0.677) | 0.630 (0.616, 0.643) |
| Hyperuricemia |  |  |  |
| BMI | 0.668 (0.639, 0.696) | **0.636 (0.616, 0.656)** | **0.605 (0.588, 0.622)** |
| WC | **0.670 (0.641, 0.699)** | 0.621 (0.601, 0.642) | 0.595 (0.578, 0.613) |
| WHtR | 0.660 (0.631, 0.689) | 0.621 (0.601, 0.641) | 0.595 (0.577, 0.612) |
|  |  |  |  |
| **Women** |  |  |  |
| Hypertension |  |  |  |
| BMI | **0.730 (0.703, 0.757)** | **0.661 (0.650, 0.672)** | **0.638 (0.626, 0.651)** |
| WC | 0.694 (0.666, 0.722) | 0.644 (0.633, 0.656) | 0.625 (0.612, 0.637) |
| WHtR | 0.712 (0.684, 0.740) | 0.649 (0.638, 0.660) | 0.630 (0.617, 0.642) |
| Diabetes |  |  |  |
| BMI | 0.744 (0.669, 0.820) | 0.640 (0.622, 0.658) | 0.618 (0.603, 0.632) |
| WC | **0.788 (0.731, 0.845)** | 0.657 (0.639, 0.674) | **0.619 (0.604, 0.633)** |
| WHtR | 0.786 (0.724, 0.848) | **0.678 (0.661, 0.695) *** | 0.618 (0.604, 0.633) |
| Dyslipidemia |  |  |  |
| BMI | **0.721 (0.691, 0.751)** | 0.603 (0.590, 0.615) | **0.563 (0.550, 0.576)** |
| WC | 0.712 (0.683, 0.741) | 0.609 (0.596, 0.622) | 0.554 (0.541, 0.567) |
| WHtR | 0.719 (0.689, 0.748) | **0.613 (0.601, 0.626)** | 0.551 (0.538, 0.564) |
| Hyperuricemia |  |  |  |
| BMI | **0.775 (0.738, 0.812)** | 0.668 (0.647, 0.689) | **0.661 (0.644, 0.678)** |
| WC | 0.738 (0.696, 0.780) | **0.677 (0.656, 0.698)** | 0.628 (0.610, 0.645) |
| WHtR | 0.738 (0.696, 0.780) | 0.674 (0.654, 0.695) | 0.629 (0.611, 0.646) |

For the definition of abbreviations, see Table 1.

**p*<0.05，(BMI *vs.* WHtR)

The figures shown in bold are the largest among the three obesity indices.

| Table S2. Optimal cut-off values for BMI, WC and WHtR that are predictive of CVD risk factors in men by age groups | | | | | | | | | | | | | | | |
| --- | --- | --- | --- | --- | --- | --- | --- | --- | --- | --- | --- | --- | --- | --- | --- |
|  |  | BMI |  |  |  |  | WC |  |  |  |  | WHtR |  |  |  |
|  | Cutoffs | Sen | Spe | YI |  | Cutoffs | Sen | Spe | YI |  | Cutoffs | Sen | Spe | YI |  |
| Hypertension |  |  |  |  |  |  |  |  |  |  |  |  |  |  |  |
| 20-44 | 24.72 | 70.06 | 64.16 | 0.34 |  | 90.10 | 42.80 | 84.65 | 0.28 |  | 0.49 | 70.06 | 62.06 | 0.32 |  |
| 45-59 | 24.39 | 68.81 | 58.29 | 0.27 |  | 84.80 | 64.79 | 59.19 | 0.24 |  | 0.51 | 65.41 | 58.88 | 0.24 |  |
| 60-74 | 23.82 | 67.39 | 55.60 | 0.23 |  | 83.95 | 62.21 | 59.54 | 0.22 |  | 0.52 | 57.53 | 64.21 | 0.22 |  |
| Diabetes |  |  |  |  |  |  |  |  |  |  |  |  |  |  |  |
| 20-44 | 23.18 | 86.11 | 38.47 | 0.25 |  | 82.32 | 77.78 | 46.89 | 0.25 |  | 0.48 | 81.94 | 46.23 | 0.28 |  |
| 45-59 | 24.83 | 63.21 | 52.51 | 0.16 |  | 89.10 | 43.87 | 72.00 | 0.16 |  | 0.51 | 62.38 | 56.30 | 0.19 |  |
| 60-74 | 24.96 | 57.97 | 58.34 | 0.16 |  | 83.95 | 66.51 | 47.45 | 0.14 |  | 0.51 | 70.49 | 43.77 | 0.14 |  |
| Dyslipidemia |  |  |  |  |  |  |  |  |  |  |  |  |  |  |  |
| 20-44 | 23.38 | 79.01 | 53.38 | 0.32 |  | 82.58 | 72.06 | 60.04 | 0.32 |  | 0.48 | 77.24 | 57.24 | 0.35 |  |
| 45-59 | 24.45 | 69.62 | 54.41 | 0.24 |  | 83.95 | 72.89 | 52.12 | 0.25 |  | 0.50 | 72.50 | 52.50 | 0.25 |  |
| 60-74 | 24.09 | 72.84 | 49.72 | 0.23 |  | 85.55 | 61.03 | 60.00 | 0.21 |  | 0.50 | 73.60 | 45.80 | 0.19 |  |
| Hyperuricemia |  |  |  |  |  |  |  |  |  |  |  |  |  |  |  |
| 20-44 | 24.91 | 62.01 | 62.93 | 0.25 |  | 84.95 | 63.20 | 61.51 | 0.25 |  | 0.50 | 60.00 | 64.50 | 0.25 |  |
| 45-59 | 24.72 | 68.93 | 51.71 | 0.21 |  | 85.58 | 65.31 | 53.18 | 0.19 |  | 0.51 | 67.51 | 50.42 | 0.18 |  |
| 60-74 | 24.71 | 60.78 | 54.62 | 0.15 |  | 83.63 | 66.72 | 46.89 | 0.14 |  | 0.52 | 62.78 | 51.48 | 0.14 |  |

For the definition of abbreviations, see Table 4.

| Table S3. Optimal cut-off values for BMI, WC and WHtR that are predictive of CVD risk factors in women by age groups. | | | | | | | | | | | | | | |
| --- | --- | --- | --- | --- | --- | --- | --- | --- | --- | --- | --- | --- | --- | --- |
|  |  | BMI |  |  |  |  | WC |  |  |  |  | WHtR |  |  |
|  | Cutoffs | Sen | Spe | YI |  | Cutoffs | Sen | Spe | YI |  | Cutoffs | Sen | Spe | YI |
| Hypertension |  |  |  |  |  |  |  |  |  |  |  |  |  |  |
| 20-44 | 22.97 | 67.81 | 66.37 | 0.34 |  | 72.90 | 74.41 | 55.13 | 0.30 |  | 0.46 | 71.50 | 59.96 | 0.32 |
| 45-59 | 24.12 | 59.21 | 64.77 | 0.24 |  | 78.68 | 60.30 | 60.78 | 0.21 |  | 0.50 | 63.58 | 58.30 | 0.22 |
| 60-74 | 24.30 | 61.10 | 58.36 | 0.20 |  | 82.90 | 55.97 | 62.87 | 0.19 |  | 0.53 | 62.13 | 57.54 | 0.20 |
| Diabetes |  |  |  |  |  |  |  |  |  |  |  |  |  |  |
| 20-44 | 24.01 | 66.67 | 73.95 | 0.41 |  | 74.58 | 82.35 | 62.09 | 0.44 |  | 0.51 | 64.71 | 81.38 | 0.46 |
| 45-59 | 24.32 | 61.92 | 58.89 | 0.21 |  | 78.96 | 69.65 | 54.08 | 0.24 |  | 0.52 | 57.54 | 68.61 | 0.26 |
| 60-74 | 24.65 | 63.92 | 53.07 | 0.17 |  | 84.90 | 55.50 | 61.79 | 0.17 |  | 0.53 | 69.07 | 47.91 | 0.17 |
| Dyslipidemia |  |  |  |  |  |  |  |  |  |  |  |  |  |  |
| 20-44 | 23.53 | 64.51 | 72.03 | 0.37 |  | 72.58 | 77.47 | 54.24 | 0.32 |  | 0.46 | 76.23 | 56.50 | 0.33 |
| 45-59 | 22.83 | 74.50 | 41.30 | 0.16 |  | 76.55 | 71.40 | 44.80 | 0.16 |  | 0.49 | 73.90 | 43.10 | 0.17 |
| 60-74 | 23.01 | 77.60 | 32.40 | 0.10 |  | 77.95 | 77.90 | 30.60 | 0.09 |  | 0.52 | 66.80 | 41.90 | 0.09 |
| Hyperuricemia | |  |  |  |  |  |  |  |  |  |  |  |  |  |
| 20-44 | 24.24 | 66.74 | 77.82 | 0.45 |  | 76.95 | 66.01 | 71.52 | 0.38 |  | 0.49 | 62.13 | 74.08 | 0.36 |
| 45-59 | 25.12 | 73.71 | 52.23 | 0.26 |  | 80.59 | 64.32 | 61.81 | 0.26 |  | 0.51 | 65.52 | 59.83 | 0.25 |
| 60-74 | 25.10 | 67.48 | 57.62 | 0.25 |  | 83.61 | 63.44 | 55.72 | 0.19 |  | 0.54 | 67.71 | 52.73 | 0.20 |

For the definition of abbreviations, see Table 4.
