# Supplementary material for: Association between anthropometric indicators of obesity and cardiovascular risk factors among adults in Shanghai, China
Source: BMC Public Health. 2019 Aug 2;19:1035. doi: 10.1186/s12889-019-7366-0 (PMC6679475; doi:10.1186/s12889-019-7366-0)
Supplement: Supplementary file 2 — Questionnaire. (DOCX 57 kb) [file 12889_2019_7366_MOESM2_ESM.docx]

*According to the provisions of Article 15 of Chapter 3 of the Statistical Law of the People's Republic of China, "the individual investigation materials belonging to the private and the family shall not be disclosed without their consent."*

**Community Residents’ Health Survey Personal Questionnaire**

| **Name of object:** **Telephone number:**  **Home address:** Songjiang District Town/Street Road  No. Room  **Zip code：**_______________ | |
| --- | --- |
|  | |
| **Start time of survey：**□□Hours□□Minutes | |
| **Signature of investigator：**  **Date：** YY MM DD | **Signature of QC clerk：**  **Date：** YY MM DD |

***School of Public Health, Fudan University***

***Songjiang District CDC, Shanghai***

***March 2016***

**Fill in the request**

1. All information is required to fill in the questionnaire by the investigator while inquiring.

2. The questionnaire must be filled with a pen, a ballpoint pen or a sigh pen, and the writing should be clear and not to be altered (if there is an alteration, the signature of the investigator is needed), the Arabic numerals must be written in regular form.

3. Circle the number of the corresponding option for the answer of the choice questions

4. The number should be filled in integer or decimal digits of the specified unit, and the "0" after the decimal cannot be omitted. For example, weight 59kg, the correct fill method is 59.00kg.

5. In addition to special instructions, you don’t need to fill any numbers or words on the logic that do not need to be investigated.

6. All time format is 24 hours.

7. Corrections after the questionnaire is filled: Use double horizontal lines on the words or numbers of the wrong item, and then fill out the correct words or numbers on the top of the line. Do not alter the original number.

8. In principle, the questions listed in the questionnaire should be investigated in sequence. Do not fill in any marks, and don't omit them.

**Informed Consent of Community Residents’ Health Survey**

Dear Sir / Madam:

With the development of social economy, improvement of living standards and accelerated process of population aging, the incidence of chronic non-communicable diseases such as diabetes mellitus and hypertension is increasing, which seriously threatens the health of the population. At present, the prevalence and causes of these chronic diseases in the community are not well understood, and the effective prevention and control measures are lacking. It has been shown that in daily life, we often be exposed to factors that may be related to these diseases. An in-depth investigation of these factors will be of great significance to the prevention and control of chronic diseases.

School of Public Health, Fudan University and Songjiang District CDC, Shanghai intend to carry out a large-scale survey of residents' health in the Songjiang community, which is a public health work for the benefit of the people and needs the participation of the whole society. Therefore, we invite you to participate in this significant work. The accuracy of the information you provide will be directly related to the success or failure of this work. In the following questionnaire, we would like to know your personal habits and health status. Please answer the questions as objectively and truthfully as possible. We will also conduct physical examination and blood biochemical test for free. We will keep the information confidential according to the relevant provisions of the statistics law of the People's Republic of China.

Thank you very much for participating in the survey. If you have any questions, please call ********.

School of Public Health, Fudan University

Songjiang District CDC, Shanghai

**Informed Consent**

I have understood the purpose of this survey and are satisfied with the introduction of the investigator. I have volunteered (or on behalf of my relatives) to participate in this investigation.

Object (or family member) Signature: Date: YY MM DD

**Investigator Statement**

I have explained the relevant matters to the survey object (Name): or his families. The object or his families have enough knowledge of this investigation and volunteered to participate in this survey.

Investigator Signature: Date: YY MM DD

**Part I Demographic Information**

**A1. Gender:** 1…Male 2…Female

**A2. Ethnic minority:** 1…Han 88…Other (________Zu)

**A3. ID number** □□□□□□□□□□□□□□□□□□

**A4. Date of birth:** YY___MM___DD

**A5. Number of family members**：

**A6. In the population of the household registration, how many people live in home in the past 6 months?** ________

**A7. Birth weight (if not clear, please fill in 99)**：_____500g

**A8. Are you a full-term product?**

1…Yes 2…No 99…Unclear

**A9. Are you raised by breastfeeding?**

1…Yes 2…No 99…Unclear

**A10. Educational level:**

1...Never gone to school 2...Primary school 3...Junior high school 4...Senior high school

5...Technical school 6... Occupation education 7... Junior College 8…undergraduate and above

**A11. Current marital status:**

1…Married 2 ... Divorced 3 ... Widowed 4 ... Single 88 ... Other

**A12. Occupation:**

1 ... Government employer 2 ... Professional and technical staff

3 ... General staff 4 ... Business / service staff

5 ... Farmer 6 ... Factory worker

7 ... Military 8 ... Student

88 ... Other, please specify

**A13. Type of Medical Insurance (multiple choice):**

1 ... Basic Medical Insurance for Urban Workers

2 ... Basic Medical Insurance for Urban Residents

3 ... New Rural Cooperative Medical Insurance

4 ... Cooperative Medical Insurance for Urban Residents

5 ... Commercial Medical Insurance:

6 ... Other Medical Insurance:

**A14. Family income last year(RMB):**

1…<10,000 2…10,000-30,000 3…31,000-50,000 4…51,000-100,000

5…100,000-150,000 6…151,000-200,000 7… 201,000-300,000 8…＞300,000

99…Unclear

**Part II Medical History and Family History**

**B1. Are you or your immediate family members diagnosed with the following diseases?**

1…Yes 2…No（jump to C1）

| **Disease**  **Category** | **Name of Disease** | **Do you have the disease？**  **(1. yes 2. no)** | **Date of first diagnosis** | **Do your family members have the disease？**  **(1. yes 2. no)** | **Number of sick family members** | | | | | |
| --- | --- | --- | --- | --- | --- | --- | --- | --- | --- | --- |
|  |  |  |  |  | Daughter (how many? __) | Son  (how many? __) | Brother  (how many? __) | Sister  (how many? __) | Father | Mother |
| **Circulatory**  **System** | **B1A1**  **Hypertension** |  | __YY__MM |  |  |  |  |  |  |  |
|  | **B1A2**  **Coronary heart disease** |  | __YY__MM |  |  |  |  |  |  |  |
|  | **B1A2_1 Type:**  1.Angina 2. Acute myocardial infarction 3. Asymptomatic 4. Ischemic cardiomyopathy 99. Unclear | | | | | | | | | |
|  | **B1A3 Stroke** |  | __YY__MM |  |  |  |  |  |  |  |
|  | **B1A3_1 Type:**  1. Cerebral hemorrhage 2. Cerebral infarction 99. Unclear | | | | | | | | | |
| **Endocrine,**  **Nutrition and**  **Metabolic** | **B1B1 Diabetes** |  | __YY__MM |  |  |  |  |  |  |  |
|  | **B1B2**  **Thyroid disease** |  | __YY__MM |  |  |  |  |  |  |  |
|  | **B1B2_1 Type:**  1. Thyroid nodules 2. Hyperthyroidism 3. Hypothyroidism 99. Unclear | | | | | | | | | |
|  | **B1B3**  **Gout**  **(Hyperuricemia)** |  | __YY__MM |  |  |  |  |  |  |  |
|  | **B1B4 Hyperlipidemia** |  | __YY__MM |  |  |  |  |  |  |  |
|  | **B1B4_1Type：**  1. hypercholesterolemia 2. Hypertriglyceridemia 3. Mixed 4. Low high-density lipoprotein 99. Unclear | | | | | | | | | |
| **Respiratory**  **System** | **B1C1 Chronic bronchitis** |  | __YY__MM |  |  |  |  |  |  |  |
|  | **B1C2 Asthma** |  | __YY__MM |  |  |  |  |  |  |  |
|  | **B1C3 COPD** |  | __YY__MM |  |  |  |  |  |  |  |
|  | **B1C4**  **Tuberculosis** |  | __YY__MM |  |  |  |  |  |  |  |
| **Digestive**  **System** | **B1D1 Gastritis** |  | __YY__MM |  |  |  |  |  |  |  |
|  | **B1D2 Enteritis** |  | __YY__MM |  |  |  |  |  |  |  |
|  | **B1D3 Intestinal polyps** |  | __YY__MM |  |  |  |  |  |  |  |
|  | **B1D4**  **Chronic hepatitis** |  | __YY__MM |  |  |  |  |  |  |  |
|  | **B1D5**  **Fatty liver** |  | __YY__MM |  |  |  |  |  |  |  |
|  | **B1D6**  **liver cirrhosis** |  | __YY__MM |  |  |  |  |  |  |  |
|  | **B1D7 Schistosomiasis** |  | __YY__MM |  |  |  |  |  |  |  |
| **Urinary**  **System** | **B1E1 Chronic kidney disease** |  | __YY__MM |  |  |  |  |  |  |  |
|  | **B1E2**  **Kidney stones** |  | __YY__MM |  |  |  |  |  |  |  |
|  | **B1E3**  **Renal cyst** |  | __YY__MM |  |  |  |  |  |  |  |
|  | **B1E4：Others ________** |  | __YY__MM |  |  |  |  |  |  |  |
| **malignant**  **tumor** | **B1F1：____________** |  | __YY__MM |  |  |  |  |  |  |  |
|  | **B1F2：____________** |  | __YY__MM |  |  |  |  |  |  |  |
|  | **B1F3：____________** |  | __YY__MM |  |  |  |  |  |  |  |

**Part III Personal Habits**

| **C1. Have you ever smoked at least one cigarette every day for more than six months?** | | | | | | |
| --- | --- | --- | --- | --- | --- | --- |
| 1…Yes→ | | **C2. How old when start smoking at least one cigarette daily?** years old | | | | |
|  | | **C3. How many cigarettes on average daily?**  branch/day | | | | |
|  | | **C4. What is your smoking status now?** | | | | |
| 2…No | | 2…No→ **C5. How old when stop smoking？** (jump to C16) | | | | |
| (jump to C16) | | 1…Yes→ | | **C6. How much do you spend on cigarettes purchase monthly?** ____yuan | | |
|  | |  | | **C7. What is the percentage of monthly cost of cigarettes for your personal monthly income?** _____% | | |
|  | |  | | **C8. Do you feel the price rose when you buy cigarettes recently?**  1…Yes 2…No 3… Unclear | | |
|  | |  | | **C9. How much does it cost to buy a pack of cigarettes you usually smoke?**  _____ yuan/package | | |
|  | |  | | **C10. How much does the price of cigarettes go up to that will reduce your smoking?**  1…___ yuan/package 2…No consideration | | |
|  | |  | | **C11. How much does the price of cigarettes go up to that will make you to consider changing the brand of cigarettes?**  1…___ yuan/package 2…No consideration | | |
|  | |  | | **C12. How much does the price of cigarettes go up to that will make you to consider giving up smoking？**  1…___ yuan/package 2…No consideration | | |
|  | |  | | **C13. Did you stop smoking for 24 hours or more to try to quit smoking in the past year?** | | |
|  | |  | | 1…Yes→ | **C14. How many times have you tried this?** | |
|  | |  | | 2…No  (jump to C16) | **C15. How long did you stop smoking for the longest time?**  **C15a**  1…day 2…week 3…month | |
|  | | | | | | |
| **D1. Have you ever been drinking alcohol at least three times a week for more than 6 months?** | | | | | | |
| 1…Yes→ | | **D2. How old when start drinking alcohol frequently?**  years old | | | | |
|  | | **D3. Have you consumed alcohol during the past year?** | | | | |
|  | | 1…Yes→ | | **D4. How many times did you drink alcohol per week in the past 12 months? (if you have been giving up drinking, please answer your drinking period)**  times | | |
|  | |  | | **D5. How much did you drink alcohol per week in the past 12 months (if you have been giving up drinking, please answer your usual weekly alcohol consumption during drinking period)？How many months do you drink alcohol like this every year?** | | |
| 2…No  (jump to E1) | | 2…No  (jump to D6) | | 1…Yellow rice wine or Rice wine D5A1 50g/week D5B1 months  2…Beer D5A2 bottle/week D5B2 months  3…Liquor D5A3 50g/week D5B3 months  4…Grape wine D5A4 50g/week D5B4 months | | |
|  |  | **D6. How old when stop drinking alcohol frequently?**  years old | | | | |

**Part Ⅳ Body Measurement**

Next, we will measure your height, weight, waist circumference and blood pressure. When measuring, please try to be as calm as possible and wear a single coat to make the measurement accurate. If you are unable to wear a single coat, please indicate your clothes in the notes. If the first two readings exceed the allowable error, please measure the third time.

| **Indicators** | **First reading** | **Second reading** | **Allowable error** | **Third reading** | **Notes** |
| --- | --- | --- | --- | --- | --- |
| **L1. height (cm)** |  |  | 1cm |  |  |
| **L2 weight (kg)** |  |  | 1kg |  |  |
| **L3 waist circumference (cm)** |  |  | 1cm |  |  |
| **L4 right arm SBP (mmHg)** |  |  | 5mmHg |  |  |
| **L5 right arm DBP(mmHg）** |  |  | 5mmHg |  |  |

**Investigators Postscript**

**M1. Whether the objects are willing to participate in the next physical examination and follow-up?**

1...Willing to 2...Don't want to

**M2. For the convenience of our follow-up, please tell us the contact address of your friends and relatives (as a contact person):**

**Name:** **Relationship with you:** **Telephone number:**

**M3. The relationship between the object of inquiry and the object of the survey?**

1. Oneself 2. Spouse 3. Parents 4. Children 5. Brother and sisters 88. Other

**M4. End time of survey:** ________ hours_____ minutes

**Thank you**
